# Supplementary material for: Laminar dynamics of high amplitude beta bursts in human motor cortex
Source: Neuroimage. 2021 Nov 15;242:118479. doi: 10.1016/j.neuroimage.2021.118479 (PMC8463839; doi:10.1016/j.neuroimage.2021.118479)
Supplement: Supplementary file 1 [file mmc1.docx]

Supplemental Figures

**
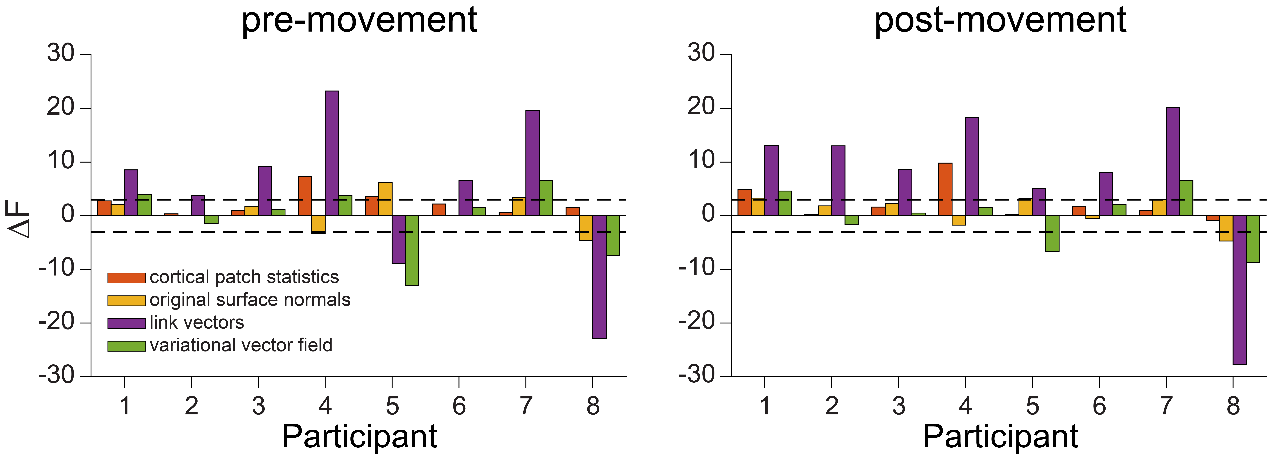
**

**Figure S1. The link vectors method for estimating cortical column orientation yields the best model fit.** Change in free energy (relative to the downsampled surface normal vectors model) for each method tested for each participant for pre-movement (left), and post-movement (right) beta bursts.

**
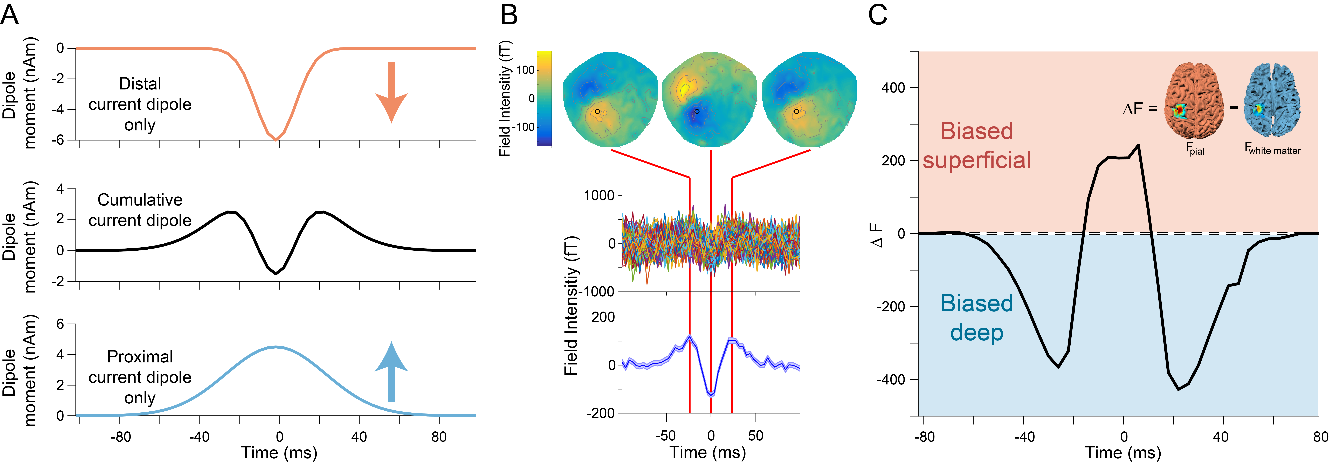
**

**Figure S2. A simple model of beta burst generation yields the same bilaminar predictions as the biophysical model.** A) In the simple model, the proximal and distal drives were modeled as Gaussian signals at oppositely oriented dipoles positioned at corresponding locations on the pial (top) and white matter (bottom) surfaces. The resulting cumulative dipole moment exchibited the same waveform features generated by the biophysical model and observed in the human MEG data (middle). B) Simulated sensor-data generated by the model has the same spatial and temporal features as beta bursts generated by the biophysical model and observed in human MEG data. C) The sliding window source inversion correctly identifies that the simulated bursts were generated by activity predominately in deep layers at the beginning and end of the burst, and predominately superficial layer activity at the peak of the burst.

**
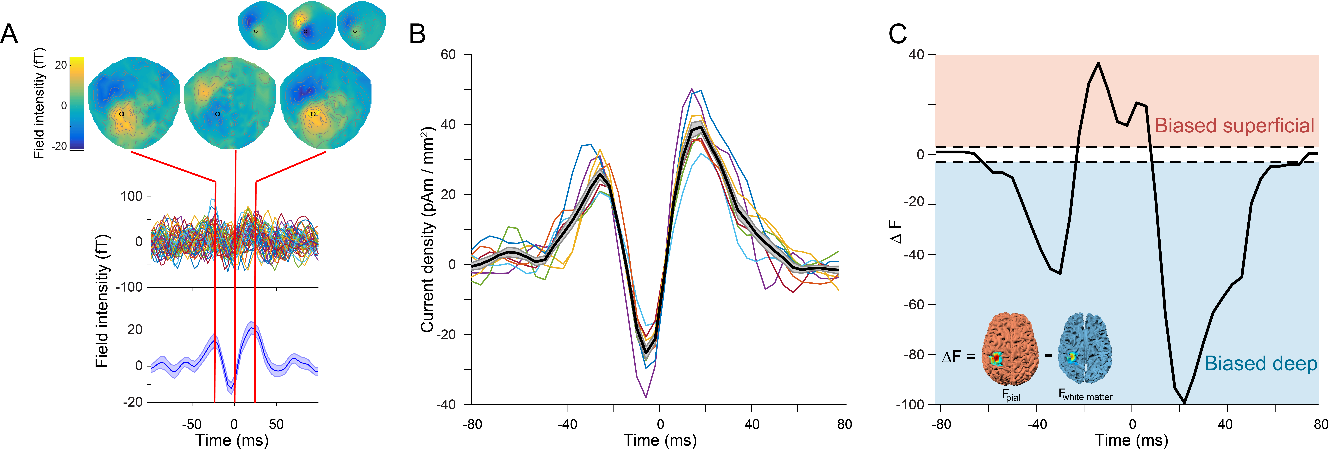
**

**Figure S3. The biophysical model with added pink noise yields the same bilaminar predictions as with white noise.** A) The biophysical model was used to generate simulated sensor level data by adding pink noise from 1-50Hz. B) Time course of source current density resulting from the localizer source inversion on the simulated sensor datasets. Each simulation (n=10) is shown as a colored line and the black line corresponds to the average over all simulations. C) The sliding window source inversion correctly identifies that the simulated bursts were generated by activity predominately in deep layers at the beginning and end of the burst, and predominately superficial layer activity at the peak of the burst.

**
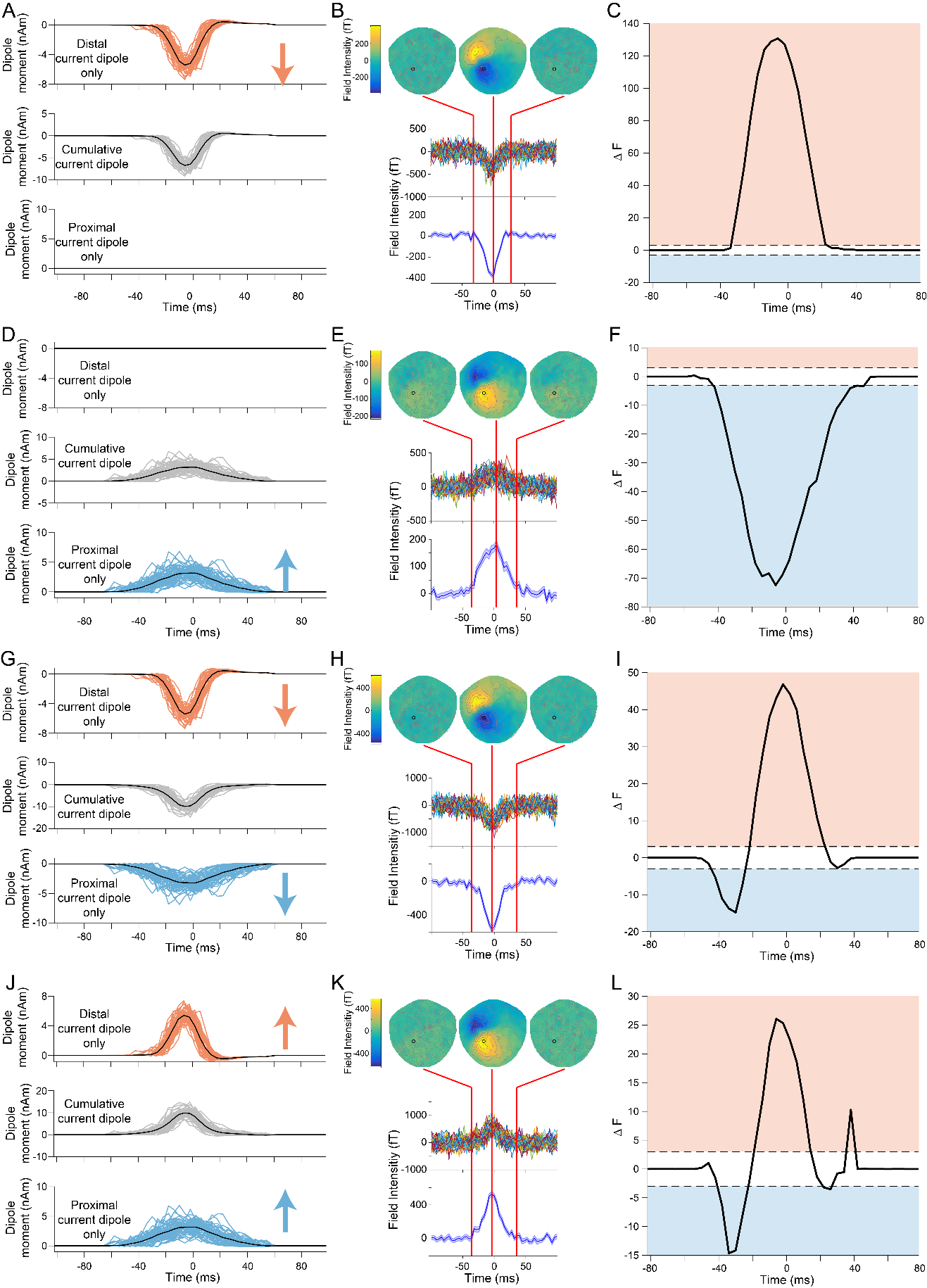
**

**Figure S4. Sliding time window source inversion can correctly identify the laminar time course of alternate synthetic models.** Alternate synthetic models including a single superficial dipole (A-C), a single deep layer dipole (D-F), a deep and superficial dipole with oriented in the direction of the deep surface (G-I), or a deep and superficial dipole oriented in the direction of the superficial surface (J-L). The sliding time window inversion correctly predicted superficial layer activity (C) and deep layer activity (F) for the single dipole models, and predominately superficial layer activity with small deep layer biases at the beginning and end of the burst for single orientation direction models (I, L).


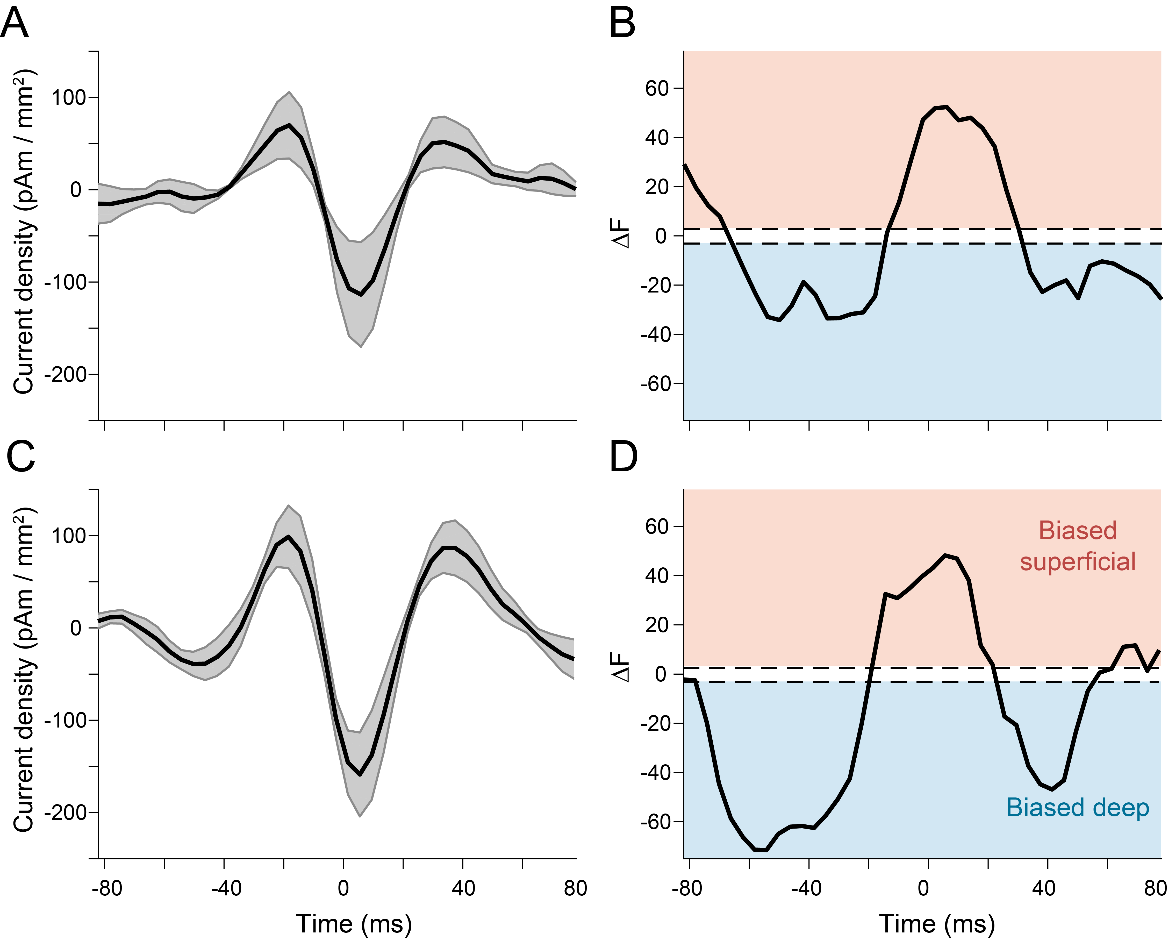


**Figure S5. Unaligned beta bursts yield the same results as aligned bursts.** A) Unaligned pre-movement beta burst source level current density time courses averaged across subjects (shaded area shows standard error). B) As with the aligned pre-movement bursts (Figure 6), activity at the beginning and end of pre-movement bursts localized to deep cortical layers, whereas activity at the peak of the bursts localized superficially. This was also true for unaligned post-movement beta bursts (C, D).

**
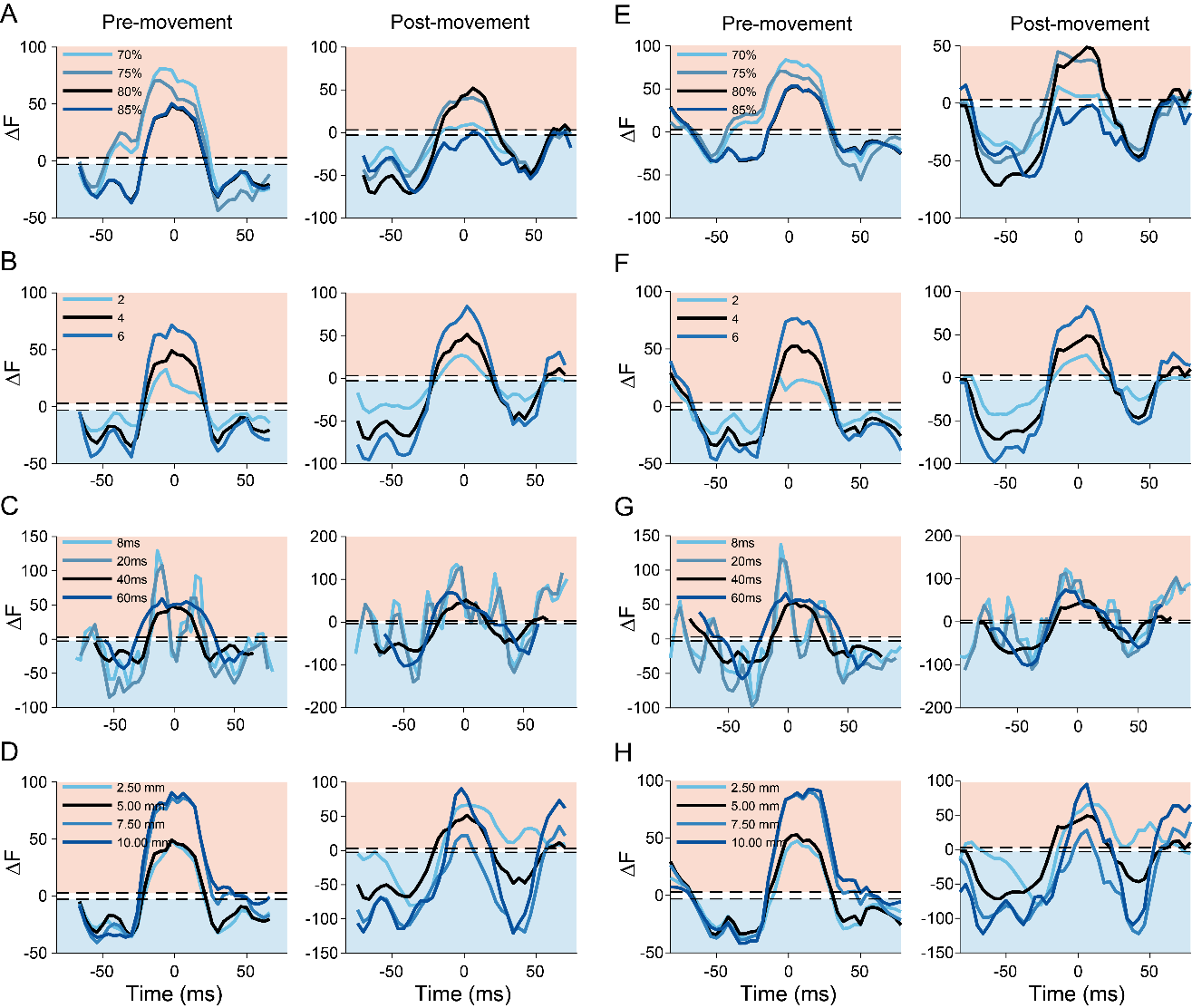
**

**Figure S6. Results bursts are robust to analysis parameters.** Results for aligned pre-movement (left column) and post-movement bursts (right column) were similar across a range of values for cluster thresholding (A), number of temporal models (B), sliding window width (C), and patch size (D). This was also true for unaligned bursts (E-H).

**
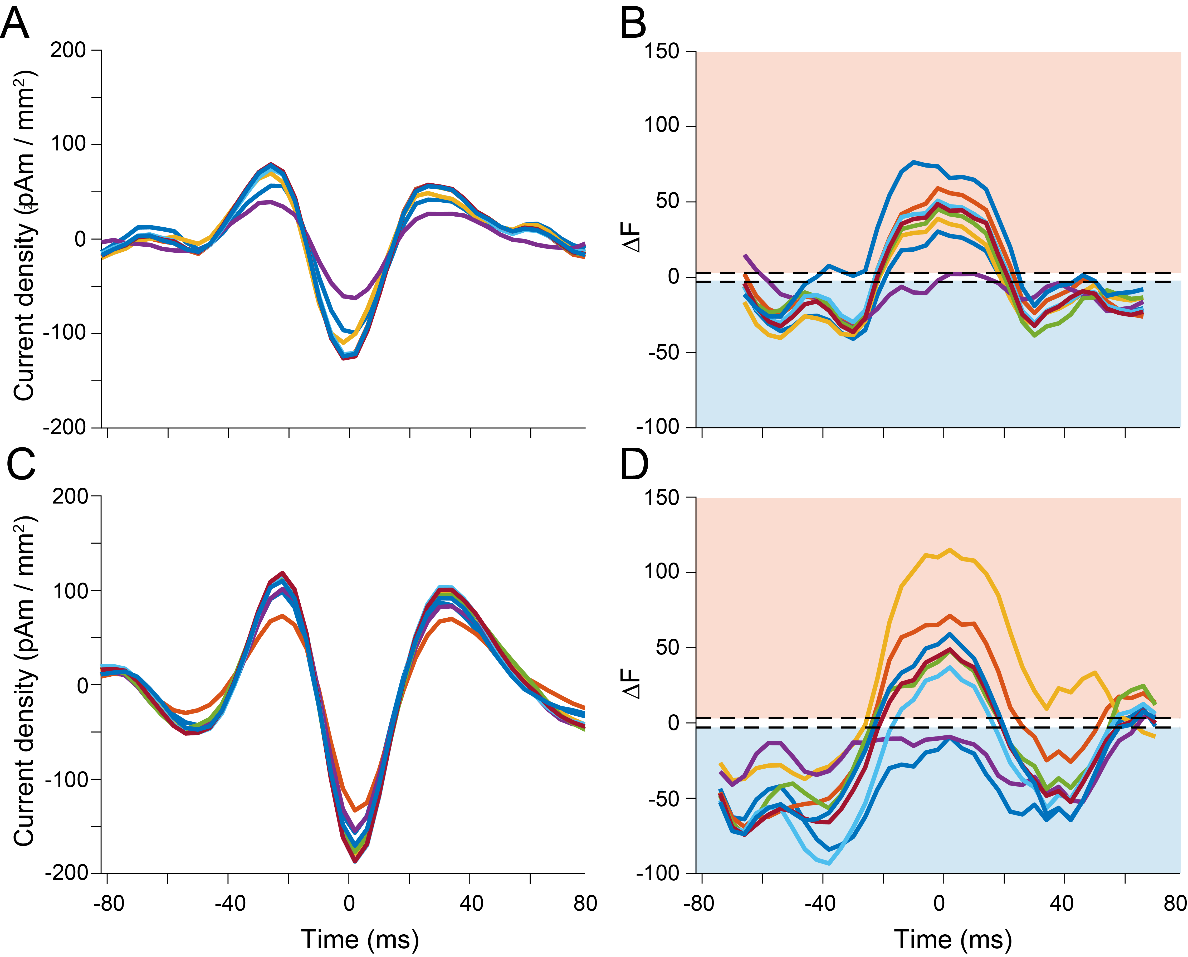
**

**Figure S7. Results are robust to individual subject differences.** A) Mean pre-movement beta burst source level current density time courses after excluding each subject (each line shows the mean after excluding a different subject). B) The time course of relatively deep or superficial activity was maintained no matter which subject was left out. This was also true for post-movement beta bursts (C, D).


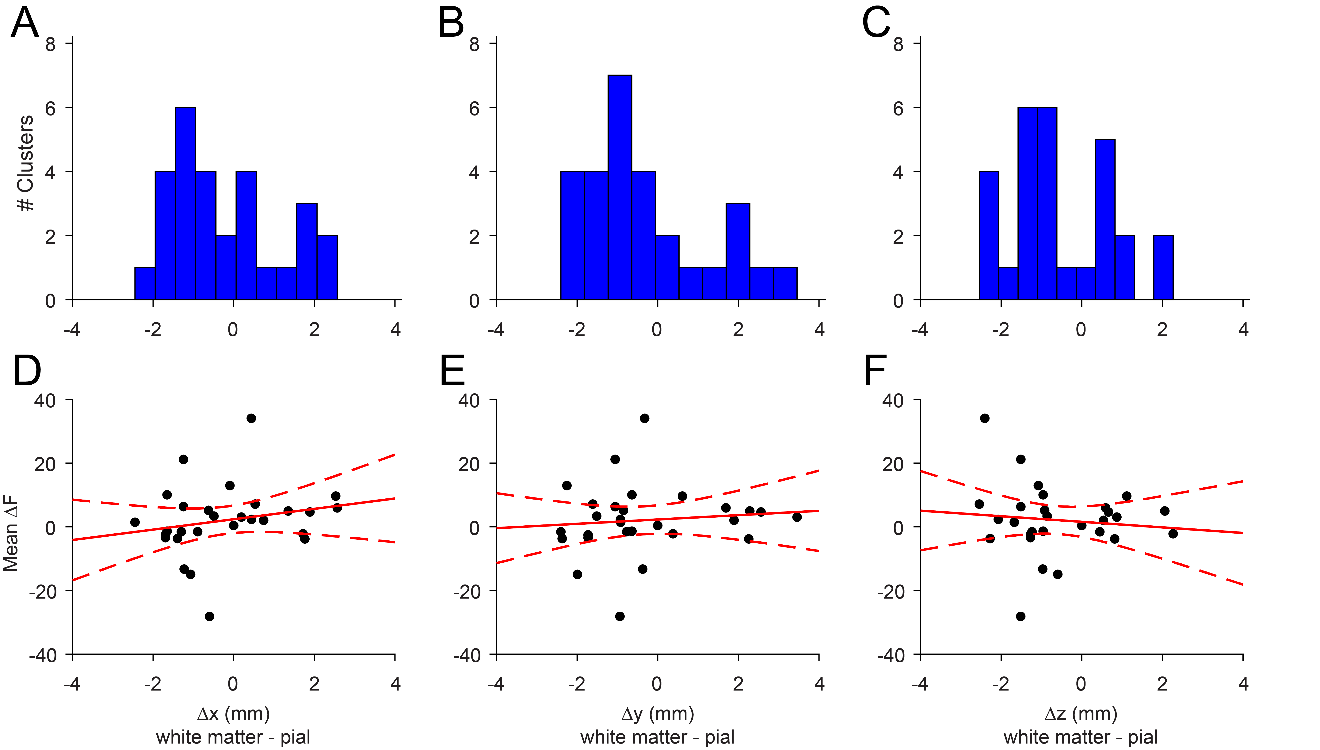


**Figure S8. *Laminar bias is not driven by cortical column orientation.*** *Top row: Distribution of white matter – pial coordinate differences in the x (left), y (middle), and z (right) dimensions over all clusters from all subjects. There is a slight bias for the deep coordinate to be posterior to the superficial coordinate (y dimension). Bottom row: Relationship between coordinate difference and laminar bias in the x (left), y (middle), and z (right) dimensions. There was no significant relationship in any dimension (x: t(26)=1.07, p=0.29; y: t(26)=0.51, p=0.61; z: t(26)=-0.53, p=0.60).*


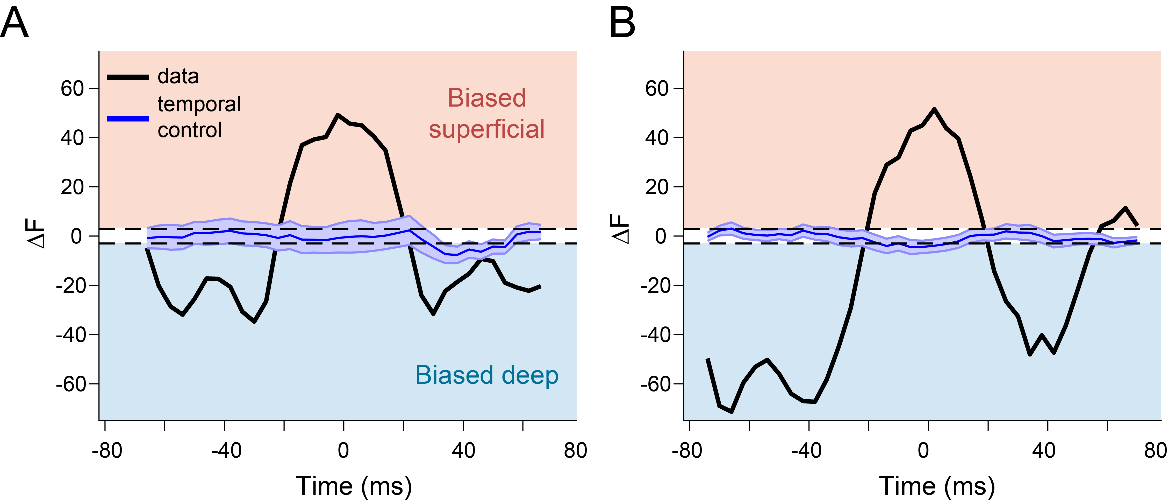


**Figure S9. Temporally shuffled surrogate data yields unbiased laminar dominance estimates.** Temporally shuffled (blue) surrogate data yields a flat Bayes factor time course that was not biased to either surface for both pre-movement (A) and post-movement (B) bursts (shaded area shows standard error).
